# Physiotherapy Management for Surgical Defect of Operated Mucormycotic Osteomyelitis of Maxilla: Post COVID-19

**DOI:** 10.7759/cureus.34733

**Published:** 2023-02-07

**Authors:** Gayatri S Kaple, Shubhangi Patil, Purva H Mundada, Nikita A Kaple

**Affiliations:** 1 Physiotherapy, Ravi Nair Physiotherapy College, Datta Meghe Institute of Medical Sciences, Wardha, IND

**Keywords:** bilateral fibrotomy, covid-19, necrosis, osteomyelitis, mucormycosis

## Abstract

The maxilla is among the jaw bones with a lot of blood flow. Maxillary bone necrosis is uncommon and can be caused by infection, trauma, or unusual metabolic abnormalities. The maxilla is a vital bone that forms the roof of the mouth cavity. *Mucormycosis* is a prevalent fungus that infects the maxilla, particularly in diabetic and immune-compromised people and post-COVID-19 patients. *Osteomyelitis* is an inflammatory condition affecting the bone and marrow tissues. It is an opportunistic infection that occurs due to the host's susceptibility to illness due to the complication of other diseases. The patient, in this case, has the chief complaint of a defect in the palate. Based on a CT brain, buccal X-Ray, and HRCT scan of Thorax. The patient's condition was diagnosed as a surgical defect in a post-operated case of mucormycotic osteomyelitis of maxilla post-COVID-19 infection. The right lower lobe's posterior segment noted a tiny soft tissue density nodule. The operated case of a surgical defect of mucormycotic osteomyelitis of maxilla post-COVID-19 infection was treated with a surgical intervention that is surgical curettage and debridement of the left maxillary sinus, bilateral fibrotomy, and reconstruction with bilateral nasolabial flap under general anesthesia. However, our objective is to enhance the quality of life, increase ventilation, increase the shoulder joint's range of motion, and enhance the joint play of the temporomandibular joint so that the patient can go back to his usual activities without difficulty. A complete pulmonary rehabilitation plan was designed to meet the patient's objectives, executed, and followed for one month. It comprised myriad interventions like bedside sitting, facial expression exercises, mouth opening exercises, neck exercises, dynamic quadriceps and hamstring, active range of motion exercises for shoulder joints, thoracic expansion exercises, and breathing exercises like diaphragmatic breathing.

## Introduction

The maxilla is the primary bone of the face that forms the upper jaw. It is a crucial viscerocranium structure that aids in creating the palate, nose, and orbit. The upper teeth are held in place by the alveolar process of the maxilla, which is vital for mastication and speaking [1]. Because of its substantial vascular supply, maxillary necrosis is uncommon compared to mandible necrosis. Maxillary necrosis might be caused by bacterial infections like osteomyelitis, viral infections like herpes zoster, fungal infections like mucormycosis, trauma, radiation, and other factors [2]. Mucormycosis usually represents an acute infection with rhino cerebral, pulmonary, gastrointestinal, cutaneous, or systemic manifestations, affecting only a small percentage of apparently healthy patients. Infection usually starts in the upper turbinates or paranasal sinuses, although it can also start in the palate or throat. In a person with poorly managed diabetes mellitus, the most common presentation in the head and neck area is maxillary and orbital cellulitis. Because mucormycosis is rare, it can be challenging to diagnose and treat for individuals unfamiliar with its clinical manifestations [3]. Osteomyelitis is a bone infection that affects the medullary cavity, Haversian system, and surrounding cortex. The maxilla is seldom involved in osteomyelitis compared to the mandible, and fungal osteomyelitis is rarely observed in the maxillofacial area [4]. Following mucormycotic infiltration into the bones, osteomyelitis develops, marked by pus production and necrosis, eventually leading to morbidity and, in some cases, death. The mandible and maxilla have been reported to be susceptible to mucormycosis. However, after COVID-19, numerous individuals developed maxillary and rhino-orbital mucormycosis.

Since the beginning of 2021, cases of mucormycosis have been documented in India as a result of COVID-19 infection [5]. However, in a post-COVID case that occurred during the COVID-19 wave, we encountered a case of mucormycotic osteomyelitis affecting the left maxilla of a 50-year-old diabetic male patient, and we present the case details and complete management of the case here with [6]. Mucormycosis has an incidence rate of 0.005 to 1.7 per million people worldwide. In India, the prevalence is 0.14 per 1000 people, which is almost 80 times greater than in wealthy countries. Mucormycosis has a global mortality rate of 46% [7]. Here, the case presentation is mucormycosis osteomyelitis of the left side's maxilla. The need to present the case is to inform the population about how the disease occurs and worsens the condition if ignored. The case report aims to establish physiotherapy management for the disease that has changed daily living to some extent [8].

## Case presentation

The present case is of 50 years old male patient, a commercial servant by occupation who had chief complaints of defects in his palate since 9 months approximately, came to tertiary care rural hospital. The patient was apparently alright nine months back when he started noticing a defect on his palate. The patient has a history of difficulty in mastication, deglutition, and speech due to a defect of the upper jaw, nasal regurgitation, and nerve paresthesia over the upper jaw region for 9 months approximately. The history patient was of COVID-19 infection in February 2021 and was hospitalized in a private hospital in Amravati for 15 days (27/02/2021) to 05/02/2021), undergoing Injection of Ramdesivir IV OD, O2 therapy, and steroid therapy. He was then diagnosed with mucormycosis of the left maxillary, ethmoidal and sphenoid sinus, for which he was hospitalized in his native place and under medication Inj. Amphotericin B IV OD 12 doses for 10 days and Tablet of Isoconazole for two months approx. The patient then reported to Tertiary Care Rural Hospital and was admitted to the oral surgery ward for further management after testing negative for COVID-19. The patient has recently been diagnosed with diabetes mellitus and was previously treated with Tablet Telma-AM 40mg OD for a 4-year history of hypertension. The patient underwent surgery under general anesthesia for surgical curettage and debridement of the left maxillary sinus, bilateral fibrotomy, and reconstruction using a bilateral nasolabial flap. The patient was then sent to the physiotherapy division for additional care.

Clinical Findings

On general examination, the patient was afebrile with a pulse rate of 80beats/minute, respiratory rate 18breaths/minute, blood pressure 124/80mmhg, height 167cm, weight 70kg, Body Mass Index (BMI) 25.10, BSA (Mosteller) 1.80, teeth 31-38, 41-48, pupils bilaterally reactive to light. On local examination, facial asymmetry is seen after previous surgery, lips competent, TMJ not clicking and deviation present with restricted mouth opening, mouth opening was 28mm approximately, teeth present 31-38, 41-48, upper edentulous arch gapping present over upper front region of palate involving anterior one-third of the palate. The patient also had pain at the operated site, which was 8/10 at the time of referral. Manual Muscle Testing (MMT) for facial muscles was also assessed. All the events that occurred during hospitalization in mentioned in (Table 1).

**Table 1:** Timeline during hospitalization.

Date of diagnosis COVID-19 positive	27/02/2021
Date of diagnosis mucormycotic osteomyelitis of the left maxilla	11/03/2021
Date of 1^st^ operation of left maxillectomy of nasal/ sphenoid exploration of mucor nasal fungal infection	17/03/2021
Date of admission to Tertiary Care Rural Hospital	07/12/2021
Date of diagnosis of surgical defect post-operated mucormycotic osteomyelitis of the maxilla	08/12/2021
Date of 2^nd^ operation of	15/12/2021
Date of referral to physiotherapy	20/12/2021
Date of discharge	27/12/2021

Diagnostic Assessment

**Figure 1:**
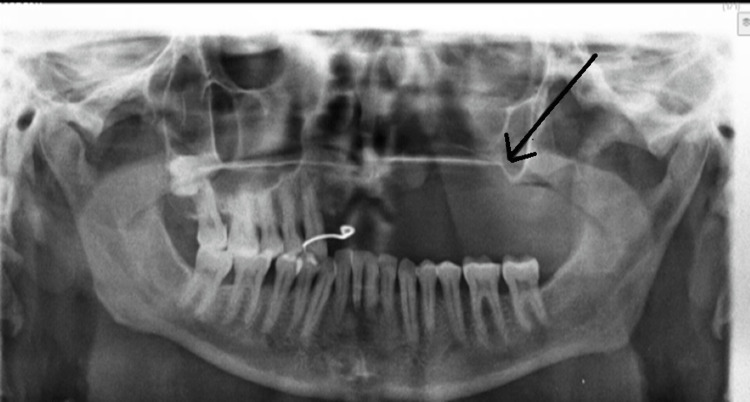
Antero-posterior view of Oral cavity showing patchy bone loss of left side of the maxilla.

Orthopantomogram: This was done in an anteroposterior view showing patchy bone loss of the maxilla of the left side (Figure 1).

CT-Paranasal Sinus (P+C): This reveals that the post-operated case of mucormycosis has mucosal thickening involving residual maxillary sinus. Mild thickening of nasopharyngeal mucosa was noted. Soft tissue extension is noted in the pterygo-maxillary and pterygoid-palatine fossa. Blood reports: Blood sugar (fasting) was 110.60mg/dl, and blood sugar (post-meal) was 154.50, more than the normal range, less than 140. HbA1c level was 6.8%, and mean blood glucose was 137.0mg%, and these results are suggestive of diabetes.

Therapeutic Intervention

Pharmacological management: Inj. Insulin Rs/s, Inj. Tramadol 50mg in 100ml NS IV stat, Inj. Emset 4mg IV stat, Inj. Clindamycin 600mg IV BD, Inj. Pan 40mg IV BD, Inj. Neomol 1mg IV BD, Inj. Perinorm 10mg IV BD, Tab. Supradyn OD, Tab. Chymoral forte BD, Tab. Levocet 10mg HS, Tab. Elastin 10mg OD, Tab. Mucinac 600mg 2 tabs TDS, Tab. Amlo 5mg OD.

Surgical management: The patient has undergone the surgery of surgical curettage and debridement of the left maxillary sinus, bilateral fibrotomy, and reconstruction with a bilateral nasolabial flap. Therefore, the patient was sedated with Midazolam and Propofol. Naso-endotracheal intubation was done from the left nostril and general anesthesia was induced. The patient was maintained on O2+N2O+Sevoflurance during the procedure of surgery. The patient was prepared and draped according to standard surgical protocol-fibrotomy of fibrous tissue bands over the right buccal mucosa. The grade of oral submucous fibrosis is stage 3(b), which is Speech and hearing deficits and the mouth opening was 24mm. Coronoidectomy has been done over the right side. Wedge-shaped white tissue was removed and sent for histopathological examination. The palatal incision was given over the maxillary region. Full-thickness mucoperiosteal flap raised. The left sinus cavity was exposed and the sinus mucosal lining of the left maxillary side was removed. Thorough debridement and curettage were done on the maxillary sinus region.

Physiotherapy Management

**Figure 2:**
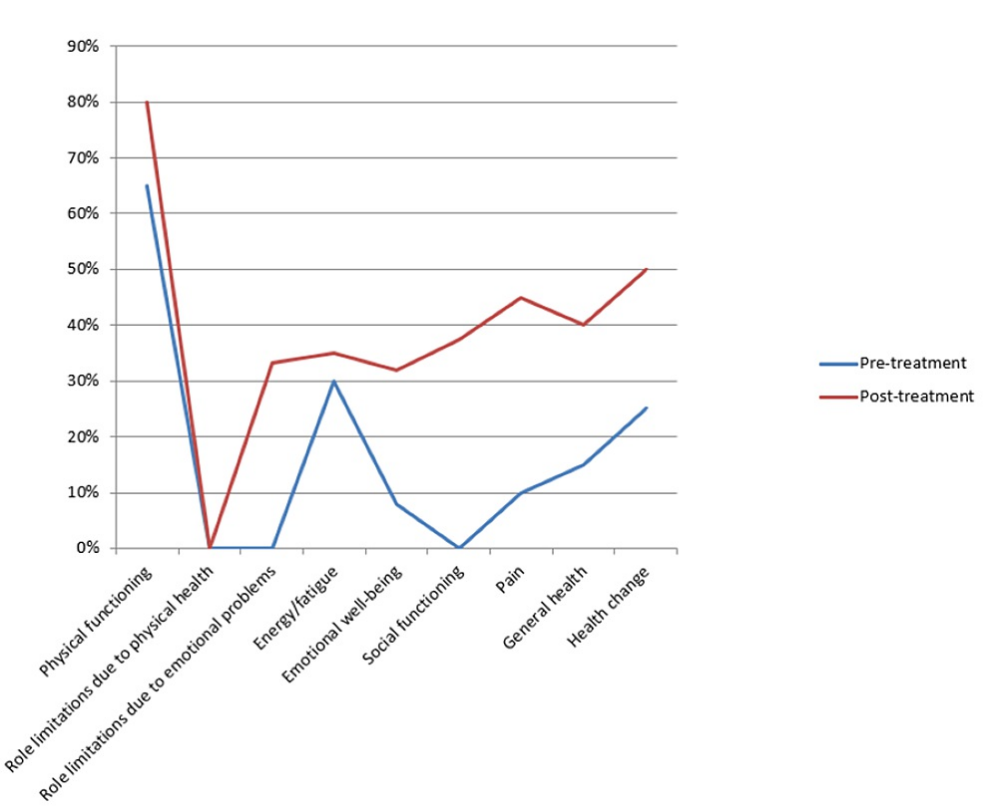
Health-Related Quality of Life SF-36

Physiotherapy interventions started targeting patients' impairment, including pain, restricted mouth opening, and lack of facial expressions (figure 2). The goals were set as follows: Physiotherapy management started early after the operation of a surgical defect in the palate. It comprised myriad interventions like Heister's mouth-opening exercises and facial expression exercises.

A treatment plan was set as per the goals, which are as follows: To decrease the pain around the incision site. To increase the mouth opening. To normalize facial expression. To increase neck and head strength. To increase the mobility of the jaw and increase the range of motion of the Temporomandibular joint.

Mouth opening by using Heister's device: On the first day of physiotherapy treatment, the patient had a mouth opening of 28mm. Therefore, the exercise was given to increase the mouth opening by using a Heister's mouth opener (Table 2). The mouth opening was maintained for 10 sec, and then the patient relaxed. The procedure was repeated for three minutes. The width and duration of the mouth opening were increased based on the improvement of the condition. Verbal commands were given to the patient. After giving mouth-opening exercises to patients daily for one month, mouth opening was improved from 28mm to 32mm.

**Table 2:** Pre-treatment and Post-treatment NPRS and Mouth opening

Outcomes	Pre-treatment	Post-treatment
Numerical Pain Rating Scale (NPRS)	8/10	6/10
Mouth opening	28mm	32mm

Facial Proprioceptive Neuromuscular Facilitation: The facial Proprioceptive Neuromuscular Facilitation protocol was added as it relies on the muscle tension created by the therapist's resistance to facial muscles, which gives a stretch to the muscle. The hold-relax technique was performed initially 15-second hold and progressed to a 30-second hold and relax. The targeting muscles include the frontalis, orbicularis oris, orbicularis oculi, buccinator, corrugator supercilious, zygomaticus, nasalis, and masseter. The patient was asked to practice these exercises for 10 repetitions, 4 times/ day respectively. The exercises were given daily for 1 month (Table 3).

**Table 3:** Strength of facial muscles after physiotherapy intervention according to sunny brook facial grading system

Facial expression	GRADE			
	Pre-treatment		Post-treatment	
	Right	Left	Right	Left
Wrinkle forehead	3	3	4	4
Close eyes normally	3	3	4	4
Close eyes forcefully	2	2	3	3
Wrinkle’s nose	1	1	2	2
Blow out cheeks	1	1	2	2
Whistle	1	1	2	2
Grin	2	2	3	3
Depress lower lip	2	2	3	3

Head-Neck Proprioceptive Neuromuscular Facilitation: An advanced chiropractic and physical therapy technique that involves both active stretching and isometric contraction of the muscle group being targeted. This improves muscle and tendon function by stimulating proprioceptive senses and enhancing muscle strength, flexibility, and balance. These exercises also increase the stability of the neck and the head.

This technique begins by placing the muscle (to be treated) in a stretched position to the point where the first slight resistance is felt. Next, the patient is instructed to resist this movement with minimum force, isometrically, for about 10 seconds and then asked to relax. 

Temporomandibular stretching: These exercises are intended to gradually restore the jaw's range of motion and reduce any discomfort caused by Temporomandibular Joint. They broadly target the jawbone at the skull's base and the throat and neck muscles. In this, there is Jaw Deviation Assisted Stretch (TMJ Deviation), Jaw Retraction Stretch (TMJ Retraction), Jaw Protrusion Stretch (TMJ Protrusion), and Temporalis Stretch. The patient is asked to perform each stretch with a 3-second hold for 10 repetitions 3 times a day.

Shoulder range of motion exercises: The has a restricted shoulder joint range of motion on the right side as the stitches are present as the flap taken is from the pectoralis muscle. The assisted range of motion exercises was given to the patient, where the patient held his right hand and took it to the range's pain-free limits.

Mouth opening exercises: Initially, the patient has a mouth opening of 28mm, so the exercise prescribed is to place your tongue against the hard palate of your mouth as your first TMJ exercise. Just below your teeth is where you will find this. Place your tongue against the hard palate behind your teeth and relax. With your lips closed, space your teeth slightly apart. Gently inhale through your nose and exhale slowly through your mouth. Hold for six seconds, and then repeat six times. Then once your mouth is open, hold your mouth open for 6 seconds [7]. Adding a slight resistance to your jaw opening and shutting will enhance muscle performance. Place two fingers on your chin and slowly open and close your lips to execute this exercise. As the patient move, the therapist's fingers should provide gentle pressure on the jaw, do not use too much effort. Six times with manual resistance, open and close your mouth, and the mouth opening has recently been improved to 32mm [8]. These exercises are prescribed for 6 repetitions 4 times/day. 

Neck exercises: Upper cervical distraction can assist in the relief of neurovascular congestion in the upper neck. This may aid in the relaxation of muscles, the reduction of tension, and the appropriate jaw movement. Neck mobilization is given for the cervical spine, and central Maitland Mobilization or Sustained Natural Apophyseal Glide is used. A single high-amplitude cervical push is used [9].

Active range of motion exercises of the shoulder joint: The shoulder scapular retraction is an effective exercise for TMJ (and increased postural awareness) for 10 repetitions 4 times/day. Capsule mobility and muscle lengthening can be improved by stretching exercises. After thorough diagnosis and assessment, do stretches for the Latissimus Dorsi, Pectoralis Major, Pectoralis Minor, Levator Scapulae, and Trapezius muscles, and teach the patient self-stretches as needed [10]. Shoulder shrugs and shoulder protraction are performed for 10 repetitions 4 times/day.

Active range of motion exercises for lower limb extremities: The exercises for lower extremities are given to mobilize the patient out of bed as soon as possible; therefore, ankle toe movements, dynamic quadriceps, dynamic hamstring, straight leg raise (SLR), pelvic bridging exercises are given for 10 repetitions each for 4 times/day.

To increase chest mobility and ventilation: Specific chest stretching and mobilization are used as part of the therapy to enhance chest mobility. When performed with relaxed breathing, thoracic expansion exercises are supposed to help loosen and evacuate pulmonary secretions, enhance collateral ventilation, and prevent fatigue, bronchospasm, and oxygen desaturation [11].

## Discussion

Mucormycosis is an opportunistic fungal illness that mainly affects immunocompromised patients, although it can also affect healthy people. Uncontrolled diabetes (especially in patients with ketoacidosis), cancers such as lymphomas and leukemias, renal failure, organ transplant, long-term corticosteroid and immunosuppressive therapy, cirrhosis, burns, protein-energy malnutrition, and acquired immune deficiency syndrome are all risk factors for mucormycosis. Uncontrolled hyperglycemia, a well-known risk factor for mucormycosis, was present in our patient [9]. In the present study, we describe a case of osteomyelitis in the left maxilla. Following the COVID-19 infection, a diabetic patient developed mucormycosis, which was documented in our research.

Regarding national prevalence, we have reported the current case of mucormycosis from India, which accounts for 72 percent of all mucormycosis cases worldwide [10]. According to the research, diabetes mellitus is a predisposing factor in 40-50 percent of mucormycosis patients [11]. Acidosis in diabetes mellitus impairs white blood cells' phagocytic capacity, compromising host immunity. Surprisingly, three of the instances discussed in this research were documented in individuals who were not immunocompromised. This conclusion was in line with our findings [12]. According to Mignogna M.D. et al., Mucormycosis in healthy people may be related to the influence of local variables in the disease's etiology. Local variables such as surgical stress from tooth extraction might affect local vascularity and serve as a gateway for bacteria to enter [13]. In a ten-year study (from January 2005 to December 2015) to assess the prevalence of fungal osteomyelitis of the jaws associated with diabetes mellitus, Niranjan et al. found that fungal osteomyelitis accounted for 52 percent of all osteomyelitis cases, while nonfungal osteomyelitis accounted for 48 percent [14]. The patient was very well trained with the exercises prescribed and felt the changes in certain things that physiotherapy intervention helped in daily activities, such as mouth opening and shoulder range improvement. In the same study, they discovered that fungal osteomyelitis is more common in men than in women above 40 and more common in males than females. The maxilla is the most frequent jaw bone affected by fungal osteomyelitis, which is more typically linked to diabetes mellitus [15]. In the present case of a diabetic patient with mucormycotic osteomyelitis of the left side of the maxilla reconstructing the surgical defect has been referred to physiotherapy after the operation; the physiotherapy management protocol is being planned to improve the quality of life of the patient and to make patient mobilize as soon as possible [16]. The protocol for this patient is mouth opening exercises, neck exercises (cervical mobilization), active shoulder range of motion, improved ventilation, and chest mobility exercises to prevent the accumulation of secretions in the chest due to infection [17].

## Conclusions

The physiotherapy rehabilitation program has been proven successful and linked to clinical significance in increasing the patient's quality of life and self-independence. This case study evaluates the integrated rehabilitation protocol for mucormycotic osteomyelitis on the left side of the maxilla. Most of the patient's recovery was not achieved during the rehabilitation program. However, all the therapeutic objectives were achieved during this plan, such as mouth opening, neck pain, early bed mobility, prevention of accumulation of secretions, and chest mobility.
